# Quality of Opioid Use Disorder Treatment for Persons With and Without Disabling Conditions

**DOI:** 10.1001/jamanetworkopen.2023.2052

**Published:** 2023-03-08

**Authors:** Cindy Parks Thomas, Maureen T. Stewart, Emily Ledingham, Rachel Sayko Adams, Lee Panas, Sharon Reif

**Affiliations:** 1 Heller School for Social Policy and Management, Brandeis University, Waltham, Massachusetts; 2 Department of Health Law, Policy and Management, Boston University School of Public Health, Boston, Massachusetts; 3 Rocky Mountain Mental Illness Research Education and Clinical Center, Veterans Health Administration, Aurora, Colorado

## Abstract

**Importance:**

Adverse outcomes associated with opioid use disorder (OUD) are disproportionately high among people with disabilities (PWD) compared with those without disability. A gap remains in understanding the quality of OUD treatment for people with physical, sensory, cognitive, and developmental disabilities, specifically regarding medications for OUD (MOUD), a foundation of treatment.

**Objective:**

To examine the use and quality of OUD treatment in adults with diagnosed disabling conditions, compared with adults without these diagnoses.

**Design, Setting, and Participants:**

This case-control study used Washington State Medicaid data from 2016 to 2019 (for use) and 2017 to 2018 (for continuity). Data were obtained for outpatient, residential, and inpatient settings with Medicaid claims. Participants included Washington State full-benefit Medicaid enrollees aged 18 to 64 years, continuously eligible for 12 months, with OUD during the study years and not enrolled in Medicare. Data analysis was performed from January to September 2022.

**Exposures:**

Disability status, including physical (spinal cord injury or mobility impairment), sensory (visual or hearing impairments), developmental (intellectual or developmental disability or autism), and cognitive (traumatic brain injury) disabilities.

**Main Outcomes and Measures:**

The main outcomes were National Quality Forum–endorsed quality measures: (1) use of MOUD (buprenorphine, methadone, or naltrexone) during each study year and (2) 6-month continuity of treatment (for those taking MOUD).

**Results:**

A total of 84 728 Washington Medicaid enrollees had claims evidence of OUD, representing 159 591 person-years (84 762 person-years [53.1%] for female participants, 116 145 person-years [72.8%] for non-Hispanic White participants, and 100 970 person-years [63.3%] for participants aged 18-39 years); 15.5% of the population (24 743 person-years) had evidence of a physical, sensory, developmental, or cognitive disability. PWD were 40% less likely than those without a disability to receive any MOUD (adjusted odds ratio [AOR], 0.60; 95% CI, 0.58-0.61; *P* < .001). This was true for each disability type, with variations. Individuals with a developmental disability were least likely to use MOUD (AOR, 0.50; 95% CI, 0.46-0.55; *P* < .001). Of those using MOUD, PWD were 13% less likely than people without disability to continue MOUD for 6 months (adjusted OR, 0.87; 95% CI, 0.82-0.93; *P* < .001).

**Conclusions and Relevance:**

In this case-control study of a Medicaid population, treatment differences were found between PWD and people without these disabilities; these differences cannot be explained clinically and highlight inequities in treatment. Policies and interventions to increase MOUD access are critical to reducing morbidity and mortality among PWD. Potential solutions include improved enforcement of the Americans with Disabilities Act, workforce best practice training, and addressing stigma, accessibility, and the need for accommodations to improve OUD treatment for PWD.

## Key Points

**Question:**

How do quality indicators for opioid use disorder (OUD) treatment for people with common physical, sensory, cognitive, or developmental disabilities compare with OUD treatment for people without these disabilities?

**Findings:**

In this case-control study of 84 728 adults with OUD enrolled in Medicaid, people with a disability were 40% less likely than people without disability to initiate medication for OUD (MOUD), with variation by disability type, and were 13% less likely to continue MOUD for 6 months.

**Meaning:**

These findings suggest that people with disability are less likely than those without disability to receive and continue taking MOUD, and addressing the large gap in MOUD initiation could improve treatment inequities.

## Teaser

This case-control study examines the use and quality of treatment for opioid use disorder in adults with diagnosed disabling conditions, compared with adults without these diagnoses.

## Introduction

Although the US federal government declared the opioid crisis a public health emergency in 2017, there were more than 100 000 drug overdose deaths in 2021, with more than 70% involving opioids.^1^ Medications to treat opioid use disorder (OUD) are effective, yet underused.^2,3^ Studies^4,5,6,7^ have revealed inequities in OUD treatment by race, ethnicity, gender, and socioeconomic status, but only limited investigation regarding access to or quality of OUD treatment is specific to people with disabilities (PWD). PWD account for 26% of the US population,^8^ and health equity is a demonstrated concern, because PWD experience barriers to health care overall.^9,10^

Importantly, PWD are at heightened risk of OUD. They are more likely to experience acute and chronic pain,^11,12^ which have been key factors associated with prescription opioid use in the US. In addition, PWD have increased risk factors for substance use disorders (SUDs), such as higher rates of mental disorders and adverse social determinants of health,^13^ and increased risk of opioid-related consequences and overdose deaths.^14,15^ However, PWD may misuse prescription opioids to relieve pain, suggesting that they are receiving inadequate pain management.^16^ Certain PWD subgroups have higher risk of opioid misuse and opioid-related consequences,^16,17,18^ although findings are not consistent.^17^ People with a history of traumatic brain injury (TBI), who are more likely to receive opioids than those without TBI, have greater risk for opioid misuse and overdose.^19^ Despite these reports, research examining OUD treatment is lacking for PWD.

Three medications for OUD (MOUD) are approved by the US Food and Drug Administration (buprenorphine, methadone, and naltrexone). Buprenorphine and methadone are associated with reduced risk of overdose compared with only psychosocial interventions, but naltrexone is not.^3^ However, MOUD is underused. A study^2^ of Medicaid enrollees in 11 states found that only 55% of individuals with OUD were receiving MOUD; adults enrolled in Medicaid because of categorical disability were less likely to be receiving MOUD than adult Medicaid enrollees without disability. Regardless of disability status, less than 60% of those receiving MOUD continued treatment for at least 6 months,^2^ a recommended minimum period demonstrating continuity and an important quality indicator.^20^ These findings reveal inequities in MOUD use for persons enrolled in Medicaid because of disability; however, we do not know whether inequities persist for a broader group of adults with disabling conditions as diagnosed by clinicians.

Barriers to OUD treatment are common, and many people with OUD are not treated.^5^ PWD experience additional barriers to SUD treatment, including stigma, inaccessible facilities and materials, difficulty accessing reliable transportation, and lack of staff disability training.^21,22^ Each type of Food and Drug Administration–approved MOUD has different requirements for use (eg, methadone generally requires daily in-person dispensing, whereas buprenorphine is usually prescribed)^3^; thus, each may present unique barriers for PWD that affect use and continuity.

A gap in knowledge remains regarding inequities in initiating and continuing MOUD treatment by disability status, key quality indicators of OUD treatment. This case-control study examines MOUD use and continuity, by disability status and type, among Medicaid-enrolled adults with OUD in Washington State compared with Medicaid-enrolled adults with no evidence of the specified disability. Administrative claims data allow examination of treatment differences between individuals who have been identified by clinicians as having a disability and those without such evidence. The study’s findings are of critical importance to clinicians, policy makers, and people with co-occurring disability and OUD, to improve health and reduce inequities and OUD-related consequences for PWD.^3^

## Methods

### Data and Study Population

 The study was approved by the Brandeis University institutional review board and was deemed exempt for the need for informed consent by the Washington State Department of Social and Health Services institutional review board because the data were deidentified, in accordance with 45 CFR §46. The report follows the Strengthening the Reporting of Observational Studies in Epidemiology (STROBE) reporting guideline for case-control studies. We examined Medicaid outpatient, inpatient, residential, and pharmacy claims from Washington State for 2016 to 2019. Using a clinically driven approach, we captured people who may not have been administratively deemed to have a disability for access to income supports and Medicaid, but who had reached a clinical threshold for diagnosis. Washington is a Medicaid expansion state, with a full continuum of SUD care, including all types of MOUD. For each year, we included adults aged 18 to 64 years with an OUD diagnosis who were continuously eligible for full Medicaid benefits for 12 months, to observe full service use. We defined OUD as at least 1 claim for outpatient, inpatient, or residential services with an OUD diagnosis code in a calendar year (eTable 1 in Supplement 1). We excluded 2587 people eligible for both Medicare and Medicaid because Medicare services were unobservable, 1132 people with a benzodiazepine prescription during the same year because concurrent MOUD is associated with increased risk of adverse effects,^23^ and 132 people because prescription days supply was missing (eFigure in Supplement 1). Analyses of MOUD treatment continuity required continuous 6 months of data following the first evidence of MOUD, requiring a look back for claims occurring in the first week of the year to see whether they were the end of an earlier episode.

### Outcome Measures

We examined use and continuity of MOUD treatment according to National Quality Forum (NQF)–endorsed measures, which many states use for Medicaid monitoring and reporting.^24^ Any MOUD use during a calendar year for patients with OUD (NQF No. 3400) is defined as having at least 1 MOUD claim during the year, overall and by type of medication.^25^ The 3 types of MOUD were identified using prescription drug data and medical procedure codes (eTable 2 in Supplement 1). The continuity measure represents continuous medication treatment for at least 6 months for those patients using MOUD (NQF No. 3175).^20^ Continuity was defined as evidence of MOUD use for 6 months after an index MOUD claim, without a 7-day gap. Full specifications for utilization and continuity are included in the eAppendix in Supplement 1.

### Disability and Types of Disability

This study used diagnosis codes to identify 4 common types of disabling conditions: physical (eg, spinal cord injuries and mobility impairment), sensory (eg, blind or visual impairments, and deaf or hard of hearing), developmental (eg, Down syndrome, autism, and other intellectual or developmental disabilities), and cognitive (ie, TBI). See eTable 3 in Supplement 1 for diagnostic codes. These conditions were informed by the US Census Bureau’s American Community Survey standardized disability types^26^ and by their association with increased risk for SUD. Each disability type was indicated through a dichotomous variable. A summary variable indicated the presence of any of these disabling conditions. Because a considerable body of research focuses on OUD treatment for individuals with co-occurring substance use and mental disorders, we did not examine these groups separately.

### Statistical Analysis

Data analysis was performed from January to September 2022. Analyses were conducted by person-year. After conducting descriptive analyses and χ^2^ tests, we fit generalized estimating equations with robust SEs assuming an exchangeable correlation structure using SAS statistical software version 9.4 (SAS Institute) to account for correlated outcomes of individuals in the data for multiple years. Two-tailed *P* < .05 was considered to denote statistical significance. Models adjusted for the confounding variables of age, gender, race and ethnicity (identified via database; race and ethnicity were included to isolate their associations with disability to the extent possible), urban residence, SUD other than OUD in the year, mental disorder in the year, eligibility year, and living in an institution for at least 2 months of the year, to be consistent with the literature. Model 1 included any disability as a dichotomous independent variable. Model 2 included each type of disability, also dichotomous with each compared with persons without that disability, as some enrollees have more than 1 type. Full models are in eTables 4, 5, 6, and 7 in Supplement 1. We also modeled type of medication by disability status and type among those receiving buprenorphine or methadone.

## Results

The sample for MOUD use analyses included 84 728 people, representing 159 591 person-years (84 762 person-years [53.1%] for female participants, 116 145 person-years [72.8%] for non-Hispanic White participants, and 100 970 person-years [63.3%] for participants aged 18-39 years). Table 1 shows sample characteristics overall and by any disability. People with the included disabling conditions represented 24 743 person-years (15.5%). Nearly half of the disability sample had a cognitive disability or TBI (11 834 person-years [47.8%]), and more than one-quarter had a physical (7304 person-years [29.5%]) or sensory (6562 person-years [26.5%]) disability. Compared with people without disability, PWD were older and more often had a mental disorder.

**Table 1.** Washington State Medicaid Enrollees With OUD, 2016-2019, by Disability Status

Characteristic	Person-years, No. (%)^a^			*P* value^b^
	Total (N = 159 591)	No disability (n = 134 848)	Any disability (n = 24 743)	
Any disability	24 743 (15.5)	NA	NA	NA
Disability type (not mutually exclusive)				
Physical	7304 (4.6)	NA	7304 (29.5)	NA
Sensory	6562 (4.1)	NA	6562 (26.5)	NA
Developmental	3124 (2.0)	NA	3124 (12.6)	NA
Cognitive	11 834 (7.4)	NA	11 834 (47.8)	NA
Age category, y				
18-29	53 144 (33.3)	47 448 (35.2)	5696 (23.0)	<.001
30-39	47 826 (30.0)	41 561 (30.8)	6265 (25.3)	
40-49	27 364 (17.1)	22 185 (16.5)	5179 (20.9)	
50-64	31 246 (19.6)	23 648 (17.5)	7598 (30.7)	
Missing	11 (<0.1)	6 (<0.1)	5 (<0.1)	
Gender				
Female	84 762 (53.1)	71 828 (53.3)	12 934 (52.3)	.01
Male	74 502 (46.7)	62 795 (46.6)	11 707 (47.3)	
Missing	327 (0.2)	225 (0.2)	102 (0.4)	
Race and ethnicity				
Hispanic	13 377 (8.4)	11 508 (8.5)	1869 (7.6)	<.001
Non-Hispanic Black	8938 (5.6)	7384 (5.5)	1554 (6.3)	
Non-Hispanic Native American	12 149 (7.6)	10 496 (7.8)	1653 (6.7)	
Non-Hispanic White	116 145 (72.8)	97 839 (72.6)	18 306 (74.0)	
Other^c^	8982 (5.6)	7621 (5.7)	1361 (5.5)	
Comorbid conditions (same year)				
Mental health disorder	96 972 (60.8)	78 123 (57.9)	18 849 (76.2)	<.001
Substance use disorder (other than OUD)	97 947 (61.4)	82 612 (61.3)	15 335 (62.0)	.03
Institution for >2 mo of year, yes	4825 (3.0)	2851 (2.1)	1974 (8.0)	<.001
Person-year of OUD diagnosis				
2016	37 676 (23.6)	31 668 (23.5)	6008 (24.3)	.007
2017	39 200 (24.6)	32 844 (24.4)	6356 (25.7)	<.001
2018	41 297 (25.9)	34 925 (25.9)	6372 (25.8)	.63
2019	41 418 (26.0)	35 411 (26.3)	6007 (24.3)	<.001
Enrollee geographic location				
Rural	21 445 (13.4)	18 319 (13.6)	3126 (12.6)	<.001
Urban	138 131 (86.6)	116 516 (86.4)	21 615 (87.4)	

Abbreviations: NA, not applicable; OUD, opioid use disorder.

^a^ Data are from 84 728 people.

^b^ *P* value for difference between no disability and any disability.

^c^ Other refers to Asian, Pacific Islander, any other race not otherwise specified, or unknown.

Unadjusted MOUD use was lower for PWD than for people without disability (9438 person-years [38.1%] vs 73 778 person-years [54.7%]) (Table 2). Unadjusted MOUD rates were lowest for people with physical (2271 person-years [31.1%]) or developmental (1027 person-years [32.9%]) disability and highest for people with cognitive disability (5075 person-years [42.9%]), but all were lower than for individuals with no disability. Both buprenorphine and methadone were less often prescribed or administered for PWD than for those without disability (buprenorphine, 4608 person-years [18.6%] vs 36 865 person-years [27.3%]; methadone, 3634 person-years [14.7%] vs 29 381 person-years [21.8%]). Naltrexone use was low in both populations, at 2.2% (534 PWD and 2905 people without disability). Rates of buprenorphine and methadone use were similar, ranging between 13.0% and 15.7%, except for people with cognitive disability, who used buprenorphine more often than methadone (2578 person-years [21.8%] vs 1777 person-years [15.0%]). Over time, unadjusted MOUD use increased overall from 39.2% (14 757 person-years) in 2016 to 64.1% (26 560 person-years) in 2019, but the difference between PWD and persons without disability remained consistent and significant (Figure).

**Table 2.** MOUD Use Among Washington State Medicaid Enrollees With OUD, by Disability Status and Disability Type, 2016-2019

Type of MOUD use	Person-years, No. (%)^a^					
	Disability status		Disability type			
	No disability (n = 134 848)	Any disability (n = 24 743)	Physical (n = 7304)	Sensory (n = 6562)	Developmental (n = 3124)	Cognitive (n = 11 834)
Any MOUD	73 778 (54.7)	9438 (38.1)	2271 (31.1)	2241 (34.2	1027 (32.9	5075 (42.9)
Buprenorphine	36 865 (27.3)	4608 (18.6)	1090 (14.9)	1032 (15.7)	467 (15.0)	2578 (21.8)
Methadone	29 381 (21.8)	3634 (14.7)	949 (13.0)	971 (14.8)	406 (13.0)	1777 (15.0)
Naltrexone	2905 (2.2)	534 (2.2)	100 (1.4)	122 (1.7)	74 (2.4)	316 (2.7)
>1 Type of MOUD	4627 (3.4)	662 (2.7)	132 (1.8)	126 (1.9)	80 (2.6)	404 (3.4)

Abbreviations: MOUD, medication for opioid use disorder; OUD, opioid use disorder.

^a^ Data are for 159 591 person-years for 84 728 people.

**Figure.**
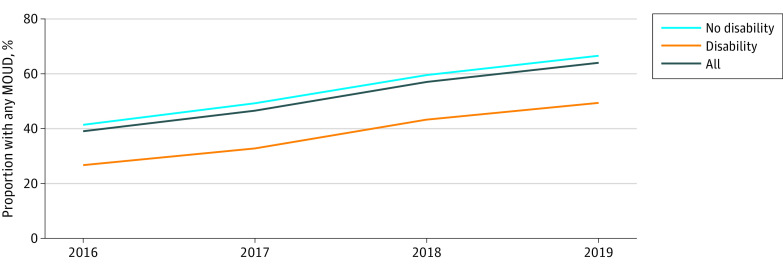
Trends in Medication for Opioid Use Disorder (MOUD) Use Over Time for Washington State Medicaid Enrollees With Opioid Use Disorder, 2016-2019, by Disability Status Graph shows unadjusted data for 84 728 people, representing 159 591 person-years.

The adjusted odds of receiving MOUD were 40% lower for PWD than for people without disability (model 1 adjusted odds ratio [AOR], 0.60; 95% CI, 0.58-0.61; *P* < .001) (Table 3). The presence of each type of disability was associated with lower MOUD use, compared with people without that disability, with differences by type of disability (model 2). Among PWD, individuals with cognitive disability were most likely to use MOUD (AOR, 0.77; 95% CI, 0.74-0.80; *P* < .001), and individuals with developmental disability had the lowest odds of MOUD use (AOR, 0.50; 95% CI, 0.46-0.55; *P* < .001). Among MOUD recipients, we found no difference in the likelihood of receiving buprenorphine vs methadone by overall disability status (eTables 8 and 9 in Supplement 1). However, individuals with developmental disability were less likely to receive buprenorphine than methadone.

**Table 3.** Continuity of MOUD 2017-2018 by Disability Status and Disability Type, Among Those With MOUD Treatment

Type of MOUD	Person-years, No. (%)^a^					
	Disability status		Disability type			
	No disability (n = 35 800)	Any disability (n = 4750)	Physical (n = 1162)	Sensory (n = 1144)	Developmental (n = 509)	Cognitive (n = 2521)
6 mo of MOUD	17 913 (50.0)	2199 (46.3)	534 (46.0)	590 (51.6)	252 (49.5)	1113 (44.2)
Buprenorphine	7100 (40.3)	834 (36.3)	188 (33.9)	215 (40.1)	97 (42.4)	441 (35.5)
Methadone	10 108 (70.8)	1249 (69.5)	324 (66.9)	350 (73.2)	144 (70.9)	600 (69.0)
Naltrexone	113 (6.9)	15 (5.1)	NA^b^	NA^b^	NA^b^	13 (7.0)
>1 Type MOUD	592 (26.1)	101 (27.9)	22 (31.9)	23 (32.9)	11 (26.2)	59 (26.3)

Abbreviations: MOUD, medication for opioid use disorder; NA, not applicable.

^a^ Data are for 40 550 person-years for 27 688 people.

^b^ Data were suppressed because of small cell sizes.

Analyses for MOUD continuity were limited to 2017 and 2018 and included 27 688 people representing 40 550 person-years. For people using MOUD, unadjusted rates (Table 3) show that PWD met the 6-month threshold for continuous MOUD less often than persons without disabilities (2199 person-years [46.3%] vs 17 913 person-years [50.0%]); those with cognitive disability had the lowest MOUD continuity rate (1113 person-years [44.2%]), and those with sensory disability had the highest continuity rate (590 person-years [51.6%]). Table 4 shows the adjusted difference in MOUD continuity by disability. People with any disability were 13% less likely than those without disability to continuously use MOUD over 6 months (model 1 AOR, 0.87; 95% CI, 0.82-0.93; *P* < .001). Individuals with a physical disability (model 2 AOR, 0.85; 95% CI, 0.74-0.96; *P* = .009) or cognitive disability (model 2 AOR, 0.89; 95% CI, 0.82-0.97; *P* = .006) were less likely than people without those conditions to continue MOUD for 6 months. Continuity did not differ for people with developmental or sensory disability vs people without those conditions.

**Table 4.** Adjusted Multivariable Analyses of MOUD Use and Continuity Among Washington State Medicaid Enrollees With OUD

Model^a^	MOUD use, 2016-2019 (n = 159 238 person-years)		MOUD 6-mo continuity, 2017-2018 (n = 40 466 person-years)	
	AOR (95% CI)	*P* value	AOR (95% CI)	*P* value
Model 1, disability status				
Any disability (reference, no disability)	0.60 (0.58-0.61)	<.001	0.87 (0.82-.93)	<.001
Model 2, disability type				
Physical (reference, no physical disability)	0.58 (0.55-0.61)	<.001	0.85 (0.74-0.96)	.009
Sensory (reference, no sensory disability)	0.61 (0.58-0.65)	<.001	0.94 (0.83-1.07)	.34
Developmental (reference, no developmental disability)	0.50 (0.46-0.55)	<.001	1.13 (0.94-1.35)	.21
Cognitive (reference, no cognitive disability)	0.77 (0.74-0.80)	<.001	0.89 (0.82-0.97)	.006

Abbreviations: AOR, adjusted odds ratio; MOUD, medication for opioid use disorder; OUD, opioid use disorder.

^a^ Models were adjusted for confounders, including age, gender, race and ethnicity, comorbid mental disorder and comorbid other substance use disorder in the year, urban vs rural location, living in an institution for more than 2 months of the year, and eligibility year.

## Discussion

The findings reported in this case-control study highlight inequities in MOUD treatment use among PWD compared with people with none of these common disabling conditions (40% less likely to use MOUD), with less disparity observed for MOUD continuity; PWD were 13% less likely than people without disability to use MOUD continuously for 6 months. These large inequities in MOUD use persisted throughout the study window and were present for each type of disabling condition examined. Differences by disability type were minor, suggesting that there are systematic barriers to initiating treatment for PWD, regardless of type of disability, compared with people without disabilities. Once taking MOUD, the differences in continuity between people with and without disability were smaller and, in some cases, insignificant. Thus, addressing inequities in initiating MOUD for PWD is most critical.

Our findings regarding MOUD use are consistent with the 11-state Medicaid study by Donohue et al,^2^ which found that individuals with OUD who were categorically eligible for Medicaid enrollment because of disability were less likely than adults without disability to use MOUD. However, the earlier study did not identify differences in continuity rates we found between PWD and people without disability, and those authors defined disability by the Medicaid categorical determination and did not consider types of disability.^2^ To our knowledge, our data are the first to examine MOUD use and continuity among persons with diagnosed potentially disabling conditions. This approach allows analysis of a larger group of patients beyond those categorically eligible for Medicaid, eliminates issues with eligibility determinations that exclude those who did not apply for or did not qualify as disabled according to employment-based guidelines, and allows a focus on disability type.^27^ We identified PWD through clinically assigned diagnoses, which factor in clinical assessments and recommendations for services. Differences by disability status in this population indicate disparities in OUD treatment quality that extend to PWD with a range of disability types. By focusing on specific types of disabilities, potential implications for policy and practice emerge.

These inequities in MOUD use and continuity cannot be explained clinically and may reflect limited access or bias in treatment approaches for PWD, which has also been identified in care not specific to OUD.^10,28^ PWD may have more medically complex conditions, yet there are no medical contraindications to using MOUD for PWD that justify this consistent disparity.^3^ Practitioners may be wary of MOUD for PWD who also have pain,^29^ and patients, in turn, may worry that OUD treatment will lead to inadequate pain management.^21,30^ To the extent that SUD treatment practitioners are unfamiliar with complex needs of PWD, practitioners may be reluctant to engage with them.^21,31^ For example, people with complex activity limitations are less likely than their peers without disability to report that practitioners listened carefully, showed respect, or explained things in an understandable way.^32^ Furthermore, only 41% of practicing US physicians reported feeling very confident about their ability to provide the same quality of care for PWD as patients without disability, and only approximately one-half reported strongly welcoming PWD into their practice.^10^ Other practitioner beliefs may play a role, such as PWD seeming to be noncompliant with treatment (vs requiring alternative means of engaging).^33^

For PWD, we found inequities in both methadone and buprenorphine use, with naltrexone used rarely. Barriers highlighted by these inequities may be related to type of MOUD,^5^ although we did not find that any 1 type of MOUD was consistently used more than another for PWD overall.

Continuity of MOUD treatment was lower for people with physical and cognitive disabilities, suggesting a potential lack of accommodations by practitioners. For people with physical disabilities, accessibility may be an ongoing burden. The finding of reduced MOUD continuity for persons with cognitive disability as defined here highlights the perfect storm model^18^ of cascading vulnerabilities for persons with TBI, for whom cognitive difficulties may lead to greater challenges in engaging with treatment.^34^ Although it might be expected that people with developmental or sensory disabilities would experience similar difficulties with ongoing treatment engagement, they had rates of MOUD continuity comparable to those for people without those conditions. This may indicate that for these individuals, once MOUD is started, remaining in treatment is less burdensome. These findings of large inequities in MOUD use and more limited inequities in continuity highlight the importance of focusing on initiating treatment as a major barrier to overcome. However, the inequities in MOUD continuity may reflect similar concerns around accessibility and stigma. Further research is needed to better understand the nuances of these findings.

Structural barriers may be substantial for PWD, regardless of MOUD type. The Americans with Disabilities Act (ADA) mandates accessibility, but not all practitioners comply.^35,36^ The last reported data,^37^ from more than a decade ago, found that many SUD treatment programs were not fully physically accessible (eg, narrow doorways or lacked ramps or elevators). Moreover, accessibility goes beyond physical means, to include offering materials and interventions that are accessible to people with visual or hearing impairments or with developmental, intellectual, or cognitive disabilities.^38^ Structural barriers imply that PWD are not welcome, and, thus, PWD may be unlikely to start treatment.

The intersecting stigma of disability and SUD heightens barriers to OUD treatment for PWD. Negative attitudes toward people with drug addiction are wide-ranging and common.^39^ PWD may experience additional challenges in seeking OUD treatment because of negative stereotypes and stigma associated with disability.^40^ Additional intersectionality (eg, race and ethnicity, gender, and mental disorders) may also play a role. Efforts to develop policies and interventions to reduce stigma and increase use of MOUD for PWD are critical to reducing future morbidity and mortality among this population, which often experiences health inequities. Efforts to improve access to MOUD should incorporate low-barrier and accessible approaches to treatment. This includes same-day treatment, wide availability of MOUD in accessible locations,^41^ and telemedicine options.^42^ Practitioner awareness of the unique challenges experienced by PWD is important, and workforce training, regarding both disability and OUD, is essential to ensuring PWD are screened for OUD and referred to MOUD. Efforts to improve access to MOUD for PWD should be implemented as part of wider efforts to educate practitioners and the community to reduce stigma around OUD and MOUD, such as through stigma reduction campaigns and engagement of regulatory and accreditation agencies.^5^

A 2-fold approach is essential for training practitioners. Practitioners who frequently engage with PWD, particularly via primary care, specialty care, and rehabilitation, should be trained to screen for OUD, be knowledgeable about MOUD treatment effectiveness, and refer patients for treatment regardless of disability status. In addition, practitioners must understand disability within their patient population and the importance of accommodations. It is essential to identify best practices for PWD specific to OUD screening and treatment, reasonable accommodation strategies, and legal obligations under the ADA. A person-centered approach is essential to reduce the inequities we observed in our study.

### Strengths and Limitations

A strength of this study is that we were able to examine types of disability and types of MOUD across a Medicaid population. However, similar to other studies using diagnosis codes to identify PWD,^17,43,44^ there are also limitations. Diagnosis-identified disabling conditions are not synonymous with functional disability^45^ but are a clinically based recognition that the patient has a potentially disabling condition that should be considered in the context of care. We examined 4 broad categories of disabling conditions; some people in the no-disability category might have these conditions but were not identified if the conditions were not recorded in the billing for a medical encounter,^27^ and some people may have other disabling conditions, making our estimates conservative. Furthermore, there are likely interaction effects of comorbidities or other factors with disability, which should be examined in further research. We chose 2 well-validated process measures of quality OUD care, but there are additional measures that may indicate disparities in care (eg, emergency department visits or opioid-related hospitalizations). We do not know patients’ history of opioid use, or whether some patients may have taken home medications. In addition, the results of this study may not be generalizable to commercially insured or Medicare populations or to Medicaid populations that are not continuously enrolled.

## Conclusions

The findings of this case-control study suggest that PWD are at greater risk of OUD than persons without disability and have more risk of SUD and adverse consequences, but are less likely to use and maintain essential treatment for OUD. Addressing the MOUD initiation gap could reduce treatment inequities. Several structural challenges exist that can be addressed by policy actions and practitioner and community education, including enforcement of ADA requirements, efforts to promote low-barrier care, and education of practitioners and community members to mitigate the heightened stigma associated with having both a disability and OUD.

## Notes

Supplement 1. **eFigure.** Study Population **eTable 1.** *ICD-9* and *ICD-10* Diagnosis and Procedure Codes Used to Identify OUD Sample **eTable 2.** OUD Medications Identified in Pharmacy and Treatment Claims **eAppendix.** Specifications for MOUD Continuity **eTable 3.** *ICD-10* Diagnosis Codes Used to Identify Disability Status and Disability Type **eTable 4.** OUD Treatment Use by Disability Status **eTable 5.** OUD Treatment Use by Disability Type **eTable 6.** 6-Month MOUD Treatment Continuity by Disability Status **eTable 7.** 6-Month MOUD Treatment Continuity by Disability Type **eTable 8.** Likelihood of Receiving Buprenorphine Versus Methadone by Disability Status **eTable 9.** Likelihood of Receiving Buprenorphine Versus Methadone by Disability Type

Supplement 2.
Data Sharing Statement
