# Supplementary material for: Quality of Opioid Use Disorder Treatment for Persons With and Without Disabling Conditions
Source: JAMA Netw Open. 2023 Mar 8;6(3):e232052. doi: 10.1001/jamanetworkopen.2023.2052 (PMC9996401; doi:10.1001/jamanetworkopen.2023.2052)
Supplement: Supplement 1. — eFigure. Study Population eTable 1. ICD-9 and ICD-10 Diagnosis and Procedure Codes Used to Identify OUD Sample eTable 2. OUD Medications Identified in Pharmacy and Treatment Claims eAppendix. Specifications for MOUD Continuity eTable 3. ICD-10 Diagnosis Codes Used to Identify Disability Status and Disability Type eTable 4. OUD Treatment Use by Disability Status eTable 5. OUD Treatment Use by Disability Type eTable 6. 6-Month MOUD Treatment Continuity by Disability Status eTable 7. 6-Month MOUD Treatment Continuity by Disability Type eTable 8. Likelihood of Receiving Buprenorphine Versus Methadone by Disability Status eTable 9. Likelihood of Receiving Buprenorphine Versus Methadone by Disability Type [file jamanetwopen-e232052-s001.pdf]

Supplemental Online Content

Thomas CP, Stewart MT, Ledingham E, Adams RS, Panas L, Reif S. Quality of opioid use disorder treatment for persons with and without disabling conditions. *JAMA Netw Open*. 2023;6(3):e232052. doi:10.1001/jamanetworkopen.2023.2052

eFigure. Study Population
eTable 1. *ICD-9* and *ICD-10 D*iagnosis and Procedure Codes Used to Identify OUD Sample
eTable 2. OUD Medications Identified in Pharmacy and Treatment Claims
eAppendix. Specifications for MOUD Continuity
eTable 3. *ICD-10* Diagnosis Codes Used to Identify Disability Status and Disability Type
eTable 4. OUD Treatment Use by Disability Status
eTable 5. OUD Treatment Use by Disability Type
eTable 6. 6-Month MOUD Treatment Continuity by Disability Status
eTable 7. 6-Month MOUD Treatment Continuity by Disability Type
eTable 8. Likelihood of Receiving Buprenorphine Versus Methadone by Disability Status
eTable 9. Likelihood of Receiving Buprenorphine Versus Methadone by Disability Type

This supplemental material has been provided by the authors to give readers additional information about their work.

© 2023 Thomas CP et al. *JAMA Network Open.*

eFigure. Study Population

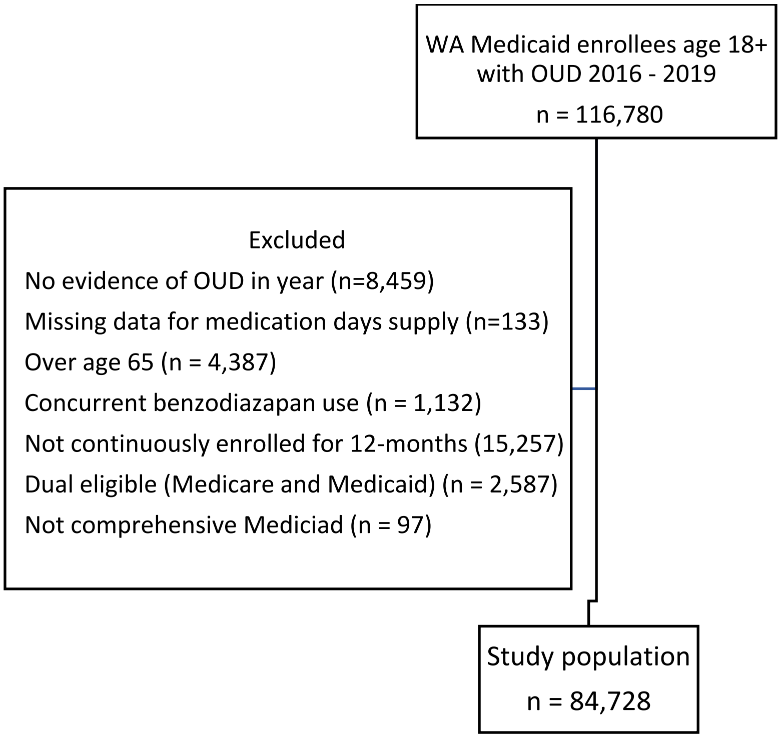

© 2023 Thomas CP et al. *JAMA Network Open.*

eTable 1. ***ICD-9*** **and** ***ICD-10 D*****iagnosis and Procedure Codes Used to Identify OUD Sample**

ICD-9 codes	3040, 3047, 3055, 9701,30400,30401,30402,30403, 30470,30471,30472,30473,30550,30551,30552,30553
ICD-10 codes	F1110, F1114, F1119, F1120, F1121, F1123, F1124, F1129, F1190, F1193, F1194, F1199, F11120, F11121, F11122, F11129, F11150, F11151, F11159, F11181, F11182, F11188, F11220, F11221, F11222, F11229, F11250, F11251, F11259, F11281, F11282, F11920, F11921, F11922, F11929, F11288, F11950, F11951, F11959, F11981, F11982, F11988,
HCPCS code	H0020

© 2023 Thomas CP et al. *JAMA Network Open.*

eTable 2. OUD Medications Identified in Pharmacy and Treatment Claims

OUD Medications
Bunavail
Buprenorphine
Buprenorphine hydrochloride
Buprenorphine-naloxone
Methadone
Naltrexone
Naltrexone hcl
Probuphine
Revia
Suboxone
Subutex
Vivitrol
Zubsolv

© 2023 Thomas CP et al. *JAMA Network Open.*

eAppendix. Specifications for MOUD Continuity

MOUD continuity is based on the National Quality Forum (NQF) measure, and is a binary variable indicating whether a person had at least 6-months of continuous MOUD treatment with less than a 7-day gap in days with medication during a year. To construct the MOUD continuity variable we first identified all MOUD covered days in 2016 - 2019 using prescription and treatment service data. We then identified the first day of medication each year for each beneficiary and looked forward to assess continuity on medication treatment. For individuals whose first evidence of medication was January 1-7 (and thus the medication day could be a continuation of an episode that was started in the previous year) we looked both forward in the current year and back to the previous year to check for evidence of a six-month continuous covered period. For individuals whose first medication day was January 8 or later we looked forward to count the number of days of continuous medication treatment in the year. For individuals whose first medication day was July 1 or later we required sufficient enrollment in the subsequent year to assess whether six months of medication coverage was met. If a beneficiary met the six-months of continuous MOUD measure at any point during the study year the measure was considered met. Due to enrollment requirements to look forward and back across years in some cases we applied this measure only to episodes in 2017 and 2018 but we used data from 2016 – 2019 to follow MOUD. The proportion of enrollees meeting the six-month continuity each year was calculated and this was used as the dependent variable for a model of continuity.

© 2023 Thomas CP et al. *JAMA Network Open.*

eTable 3. *ICD-10* Diagnosis Codes Used to Identify Disability Status and Disability Type

Disability type	ICD10 diagnosis codes
Physical	G041, G114, G8100, G8101, G8102, G8103, G8104, G8110, G8111, G8112, G8113, G8114, G8190, G8191, G8192, G8193, G8194, G8220, G8221, G8222, G8250, G8251, G8252, G8253, G8254, G830, G8310, G8311, G8312, G8313, G8314, G8320, G8321, G8322, G8323, G8324, G8330, G8331, G8332, G8333, G8334, G834, G835, G8381, G8382, G8383, G8384, G8389, G839, I69031, I69032, I69033, I69034, I69039, I69041, I69042, I69043, I69044, I69049, I69051, I69052, I69053, I69054, I69059, I69061, I69062, I69063, I69064, I69065, I69069, I69131, I69132, I69133, I69134, I69139, I69141, I69142, I69143, I69144, I69149, I69151, I69152, I69153, I69154, I69159, I69161, I69162, I69163, I69164, I69165, I69169, I69231, I69232, I69233, I69234, I69239, I69241, I69242, I69243, I69244, I69249, I69251, I69252, I69253, I69254, I69259, I69261, I69262, I69263, I69264, I69265, I69269, I69331, I69332, I69333, I69334, I69339, I69341, I69342, I69343, I69344, I69349, I69351, I69352, I69353, I69354, I69359, I69361, I69362, I69363, I69364, I69365, I69369, G710, G7100, G7101, G7102, G7109, G7111, G712, G9611, S12000A, S12001A, S12100A, S12101A, S12200A, S12201A, S12300A, S12301A, S12400A, S12401A, S12500A, S12501A, S12600A, S12601A, S129XXA, S12000B, S12001B, S12100B, S12101B, S12200B, S12201B, S12300B, S12301B, S12400B, S12401B, S12500B, S12501B, S12600B, S12601B, S140XXA, S140XXS, S14101A, S14102A, S14103A, S14104A, S14105A, S14106A, S14107A, S14108A, S14109A, S14111A, S14112A, S13113A, S14114A, S14115A, S14116A, S14117A, S14118A, S14119A, S14121A, S14122A, S14123A, S14124A, S14125A, S14126A, S14127A, S14128A, S14129A, S14131A, S14132A, S14133A, S14134A, S14135A, S14136A, S14137A, S14138A, S14139A, S14141A, S14142A, S14143A, S14144A, S14145A, S14146A, S14147A, S14148A, S14149A, S14151A, S14152A, S14153A, S14154A, S14155A, S14156A, S14157A, S14158A, S14159A, S14101S, S14102S, S14103S, S14104S, S14105S, S14106S, S14107S, S14108S, S14109S, S14111S, S14112S, S14113S, S14114S, S14115S, S14116S, S14117S, S14118S, S14119S, S14121S, S14122S, S14123S, S14124S, S14125S, S14126S, S14127S, S14128S, S14129S, S14131S, S14132S, S14133S, S14134S, S14135S, S14136S, S14137S, S14138S, S14139S, S14141S, S14142S, S14143S, S14144S, S14145S, S14146S, S14147S, S14148S, S14149S, S14151S, S14152S, S14153S, S14154S, S14155S, S14156S, S14157S, S14158S, S14159S, S22009A, S22019A, S22029A, S22039A, S22049A, S22059A, S22069A, S22079A, S22089A, S22009B, S22019B, S22029B, S22039B, S22049B, S22059B, S22069B, S22079B, S22089B, S240XXA, S24101A, S24102A, S24103A, S24104A, S24109A, S24111A, S24112A, S24113A, S24114A, S24119A, S24131A, S24132A, S24133A, S24134A, S24139A, S24141A, S24142A, S24143A, S24144A, S24149A, S24151A, S24152A, S24153A, S24154A, S24159A, S240XXS, S24101S, S24102S, S24103S, S24104S, S24109S, , S24111S, S24112S, S24113S, S24114S, S24119S, S24131S, S24132S, S24133S, S24134S, S24139S, S24141S, S24142S, S24143S, S24144S, S24149S, S24151S, S24152S, S24153S, S24154S, S24159S, S32009A, S32019A, S32029A, S32039A, S32049A, S32059A, S32009B, S32019B, S32029B, S32039B, S32049B, S32059B, S3210XA, S322XXA, S3210XB, S322XXB, S3401XA, S3402XA, S34101A, S34102A, S34103A, S34104A, S34105A, S34109A, S34111A, S34112A, S34113A, S34114A, S34115A, S34119A, S34121A, S34122A, S34123A, S34124A, S34125A, S34129A, S34131A, S34132A, S34139A, S343XXA, S3401XS, S3402XS, S34101S, S34102S, S34103S, S34104S, S34105S, S34109S, S34111S, S34112S, S34113S, S34114S, S34115S, S34119S, S34121S, S34122S, S34123S, S34124S, S34125S, S34129S, S34131S, S34132S, S34139S,
Developmental	E7871, E7872, F70, F71, F72, F73, F78, F79, P043, Q860, Q871, Q8711, Q8719, Q872, Q873, Q875, Q8781, Q8789, Q897, Q898, Q900, Q901, Q902, Q909, Q910, Q911, Q912, Q913, Q914, Q915, Q916, Q917, Q920, Q921, Q922, Q925, Q9261, Q9262, Q927, Q928, Q929, Q930, Q931, Q932, Q933, Q934, Q935, Q9351, Q93529, Q937, Q9381, Q9388, Q9389, Q939, Q952, Q953, Q992, F840, F843, F845, F848, F849, F819, F82, F88, F89,
Sensory	H540, H540X33, H540X34, H540X35, H540X43, H540X44, H540X45, H540X53, H540X54, H540X55, H5410, H5411, H541131, H541132, H541141, H541142, H541151, H541152, H5412, H541213, H541214, H541215, H541223, H541224, H541225, H542, H542X11, H542X12, H542X21, H542X22, H543, H548, H903, H9041, H9042, H905, H906, H9071, H9072, H908, H90A21, H90A22, H90A31, H90A32, H9101, H9102, H9103, H9109, H913, H918X1, H918X2, H918X3, H918X9, H9190, H9191, H9192, H9193,
Cognitive (TBI)	F0781, S060X0A, S060X0D, S060X0S, S060X1A, S060X1D, S060X1S, S060X2A, S060X2D, S060X2S, S060X3A, S060X3D, S060X3S, S060X4A, S060X4D, S060X4S, S060X5A, S060X5D, S060X5S, S060X6A, S060X6D, S060X6S, S060X7A, S060X7D, S060X7S, S060X8A, S060X8S, S060X9A, S060X9D, S060X9S, S061X0A, S061X0D, S061X0S, S061X1A, S061X1D, S061X1S, S061X2A, S061X2D, S061X2S, S061X3A, S061X3D, S061X3S, S061X4A, S061X4D, S061X4S, S061X5A, S061X5D, S061X5S, S061X6A, S061X6D, S061X6S, S061X7A, S061X7D, S061X7S, S061X8A, S061X8D, S061X8S, S061X9A, S061X9D, S061X9S, S062X0A, S062X0D, S062X0S, S062X1A, S062X1D, S062X1S, S062X2A, S062X2D, S062X2S, S062X3A, S062X3D, S062X3S, S062X4A, S062X4D, S062X4S, S062X5A, S062X5D, S062X5S, S062X6A, S062X6D, S062X6S, S062X7A, S062X7D, S062X7S, S062X8A, S062X8D, S062X8S, S062X9A, S062X9D, S062X9S, S06300A, S06300D, S06300S, S06301A, S06301D, S06301S, S06302A, S06302D, S06302S, S06303A, S06303D, S06303S, S06304A, S06304D, S06304S, S06305A, S06305D, S06305S, S06306A, S06306D, S06306S, S06307A, S06307D, S06307S, S06308A, S06308D, S06308S, S06309A, S06309D, S06309S, S06310A, S06310D, S06310S, S06311A, S06311D, S06311S, S06312A, S06312D, S06312S, S06313A, S06313D, S06313S, S06314A, S06314D, S06314S, S06315A, S06315D, S06315S, S06316A, S06316D, S06316S, S06317A, S06317D, S06317S, S06318A, S06318D, S06318S, S06319A, S06319D, S06319S, S06320A, S06320D, S06320S, S06321A, S06321D, S06321S, S06322A, S06322D, S06322S, S06323A, S06323D,

© 2023 Thomas CP et al. *JAMA Network Open.*

Disability type:	Diagnostic codes
Cognitive (TBI)	S06323S, S06324A, S06324D, S06324S, S06325A, S06325D, S06325S, S06326A, S06326D, S06326S, S06327A, S06327D, S06327S, S06328A, S06328D, S06328S, S06329A, S06329D, S06329S, S06330A, S06330D, S06330S, S06331A, S06331D, S06331S, S06332A, S06332D, S06332S, S06333A, S06333D, S06333S, S06334A, S06334D, S06334S, S06335A, S06335D, S06335S, S06336A, S06336D, S06336S, S06337A, S06337D, S06337S, S06338A, S06338D, S06338S, S06339A, S06339D, S06339S, S06340A, S06340D, S06340S, S06341A, S06341D, S06341S, S06342A, S06342D, S06342S, S06343A, S06343D, S06343S, S06344A, S06344D, S06344S, S06345A, S06345D, S06345S, S06346A, S06346D, S06346S, S06347A, S06347D, S06347S, S06348A, S06348D, S06348S, S06349A, S06349D, S06349S, S06350A, S06350D, S06350S, S06351A, S06351D, S06351S, S06352A, S06352D, S06352S, S06353A, S06353D, S06353S, S06354A, S06354D, S06354S, S06355A, S06355D, S06355S, S06356A, S06356D, S06356S, S06357A, S06357D, S06357S, S06358A, S06358D, S06358S, S06359A, S06359D, S06359S, S06360A, S06360D, S06360S, S06361A, S06361D, S06361S, S06362A, S06362D, S06362S, S06363A, S06363D, S06363S, S06364A, S06364D, S06364S, S06365A, S06365D, S06365S, S06366A, S06366D, S06366S, S06367A, S06367D, S06367S, S06368A, S06368D, S06368S, S06369A, S06369D, S06369S, S06370A, S06370D, S06370S, S06371A, S06371D, S06371S, S06372A, S06372D, S06372S, S06373A, S06373D, S06373S, S06374A, S06374D, S06374S, S06375A, S06375D, S06375S, S06376A, S06376D, S06376S, S06377A, S06377D, S06377S, S06378A, S06378D, S06378S, S06379A, S06379D, S06379S, S06380A, S06380D, S06380S, S06381A, S06381D, S06381S, S06382A, S06382D, S06382S, S06383A, S06383D, S06383S, S06384A, S06384D, S06384S, S06385A, S06385D, S06385S, S06386A, S06386D, S06386S, S06387A, S06387D, S06387S, S06388A, S06388D, S06388S, S06389A, S06389D, S06389S, S064X0A, S064X0D, S064X0S, S064X1A, S064X1D, S064X1S, S064X2A, S064X2D, S064X2S, S064X3A, S064X3D, S064X3S, S064X4A, S064X4D, S064X4S, S064X5A, S064X5D, S064X5S, S064X6A, S064X6D, S064X6S, S064X7A, S064X7D, S064X7S, S064X8A, S064X8D, S064X8S, S064X9A, S064X9D, S064X9S, S065X0A, S065X0D, S065X0S, S065X1A, S065X1D, S065X1S, S065X2A, S065X2D, S065X2S, S065X3A, S065X3D, S065X3S, S065X4A, S065X4D, S065X4S, S065X5A, S065X5D, S065X5S, S065X6A, S065X6D, S065X6S, S065X7A, S065X7D, S065X7S, S065X8A, S065X8D, S065X8S, S065X9A, S065X9D, S065X9S, S066X0A, S066X0D, S066X0S, S066X1A, S066X1D, S066X1S, S066X2A, S066X2D, S066X2S, S066X3A, S066X3D, S066X3S, S066X4A, S066X4D, S066X4S, S066X5A, S066X5D, S066X5S, S066X6A, S066X6D, S066X6S, S066X7A, S066X7D, S066X7S, S066X8A, S066X8D, S066X8S, S066X9A, S066X9D, S066X9S, S06810A, S06810D, S06810S, S06811A, S06811D, S06811S, S06812A, S06812D, S06812S, S06813A, S06813D, S06813S, S06814A, S06814D, S06814S, S06815A, S06815D, S06815S, S06816A, S06816D, S06816S, Cognitive S06817A, S06817D, S06817S, S06818A, S06818D, S06818S, S06819A, S06819D, S06819S, S06820A, S06820D, S06820S, S06821A, S06821D, S06821S, S06822A, S06822D, S06822S, S06823A, S06823D, S06823S, S06824A, S06824D, S06824S, S06825A, S06825D, S06825S, S06826A, S06826D, S06826S, S06827A, S06827D, S06827S, S06828A, S06828D, S06828S, S06829A, S06829D, S06829S, S06890A, S06890D, S06890S, S06891A, S06891D, S06891S, S06892A, S06892D, S06892S, S06893A, S06893D, S06893S, S06894A, S06894D, S06894S, S06895A, S06895D, S06895S, S06896A, S06896D, S06896S, S06897A, S06897D, S06897S, S06898A, S06898D, S06898S, S06899A, S06899D, S06899S, S069X0A, S069X0D, S069X0S, S069X1A, S069X1D, S069X1S, S069X2A, S069X2D, S069X2S, S069X3A, S069X3D, S069X3S, S069X4A, S069X4D, S069X4S, S069X5A, S069X5D, S069X5S, S069X6A, S069X6D, S069X6S, S069X7A, S069X7D, S069X7S, S069X8A, S069X8D, S069X8S, S069X9A, S069X9D, S069X9S, V1552, Z87820

© 2023 Thomas CP et al. *JAMA Network Open.*

eTable 4. OUD Treatment Use by Disability Status
Generalized linear regression analysis for the outcome OUD treatment use (n = 159,238 person-years for 83,052 people)

Analysis Of GEE Parameter Estimates						
**Empirical Standard Error Estimates**						
**Parameter**	**Estimate**	**Standard Error**	**95% Confidence Limits**		**Z**	**Pr > |Z|**
**Intercept**	-0.3176	0.0218	-0.3603	-0.2749	-14.58	<.0001
**Any Disability (ref=No disability)**	-0.5174	0.0150	-0.5469	-0.4879	-34.40	<.0001
Age (ref = 18-29)						
30-39	0.0197	0.0132	-0.0062	0.0457	1.49	0.1362
40-49	-0.3487	0.0156	-0.3793	-0.3181	-22.35	<.0001
50-64	-0.7551	0.0157	-0.7859	-0.7244	-48.14	<.0001
**Female (ref = male)**	0.0058	0.0107	-0.0152	0.0268	0.54	0.5890
Race/ Ethnicity (ref = Non-Hispanic, White)						
Non-Hispanic, Black	-0.4776	0.0240	-0.5247	-0.4305	-19.88	<.0001
Non-Hispanic, American Indian	-0.0826	0.0203	-0.1223	-0.0429	-4.08	<.0001
Hispanic	-0.2554	0.0190	-0.2926	-0.2183	-13.47	<.0001
Other/ unknown race	-0.4407	0.0232	-0.4861	-0.3952	-19.02	<.0001
Comorbidities						
Mental health (MH) (re=no MH comorbidity)	-0.1536	0.0113	-0.1757	-0.1315	-13.61	<.0001
Other substance use disorder (SUD) (ref = no other SUD)	0.2420	0.0111	0.2201	0.2638	21.73	<.0001
Eligibility Year (ref = 2016)						
2017	0.3326	0.0151	0.3029	0.3623	21.97	<.0001
2018	0.7574	0.0150	0.7280	0.7868	50.52	<.0001
2019	1.0524	0.0152	1.0227	1.0821	69.42	<.0001
**Urban (ref = rural)**	0.2155	0.0154	0.1853	0.2457	13.98	<.0001
**Institution (ref = not in institution)**	-1.1447	0.0363	-1.2159	-1.0735	-31.49	<.0001

© 2023 Thomas CP et al. *JAMA Network Open.*

eTable 5. OUD Treatment Use by Disability Type
Generalized linear regression analysis for the outcome oud treatment use (n = 159,238 person-years for 83,052 people)

Analysis Of GEE Parameter Estimates						
Empirical Standard Error Estimates						
**Parameter**	**Estimate**	**Standard Error**	**95% Confidence Limits**		**Z**	**Pr > |Z|**
**Intercept**	-0.3208	0.0218	-0.3635	-0.2781	-14.72	<.0001
Disability type						
Developmental (ref = no developmental disability)	-0.6878	0.0412	-0.7686	-0.6070	-16.68	<.0001
Physical (ref = no physical disability)	-0.5536	0.0274	-0.6073	-0.4999	-20.21	<.0001
Sensory (ref = no sensory disability)	-0.4893	0.0281	-0.5445	-0.4341	-17.39	<.0001
Cognitive (ref = no cognitive disability)	-0.2679	0.0207	-0.3086	-0.2273	-12.92	<.0001
Age *(ref = 18-29)*						
30-39	0.0206	0.0133	-0.0054	0.0466	1.56	0.1199
40-49	-0.3469	0.0156	-0.3776	-0.3162	-22.18	<.0001
50-64	-0.7489	0.0158	-0.7798	-0.7180	-47.48	<.0001
**Female (ref = male)**	0.0085	0.0107	-0.0125	0.0296	0.79	0.4269
Race/ Ethnicity (ref = Non-Hispanic, White)						
Non-Hispanic, Black	-0.4809	0.0240	-0.5280	-0.4338	-20.02	<.0001
Non-Hispanic, American Indian	-0.0833	0.0203	-0.1231	-0.0436	-4.11	<.0001
Hispanic	-0.2567	0.0190	-0.2939	-0.2195	-13.53	<.0001
Other/ unknown race	-0.4394	0.0232	-0.4848	-0.3940	-18.97	<.0001
Comorbidities						
Mental health (MH) (ref = No MH comorbidity)	-0.1534	0.0113	-0.1756	-0.1313	-13.59	<.0001
Other substance use disorder (SUD) (ref = No SUD comorbidity)	0.2369	0.0112	0.2151	0.2588	21.24	<.0001
Eligibility Year (ref = 2016)						
2017	0.3322	0.0151	0.3026	0.3619	21.94	<.0001
2018	0.7562	0.0150	0.7268	0.7856	50.43	<.0001
2019	1.0517	0.0152	1.0220	1.0814	69.34	<.0001
**Urban (ref = rural)**	0.2153	0.0154	0.1851	0.2456	13.96	<.0001
**Institution (ref = not in institution)**	-1.1194	0.0366	-1.1911	-1.0478	-30.62	<.0001

© 2023 Thomas CP et al. *JAMA Network Open.*

eTable 6. 6-Month MOUD Treatment Continuity by Disability Status
Generalized linear regression analysis for the outcome 6-month moud treatment continuity (n = 40,550 person-years for 27,688 people)

Analysis Of GEE Parameter Estimates						
**Empirical Standard Error Estimates**						
**Parameter**	**Estimate**	**Standard Error**	**95% Confidence Limits**		**Z**	**Pr > |Z|**
**Intercept**	0.3710	0.0408	0.2910	0.4510	9.09	<.0001
**Any Disability (ref = No disability)**	-0.1402	0.0327	-0.2043	-0.0762	-4.29	<.0001
Age (ref = 18-29)						
30-39	0.3264	0.0245	0.2784	0.3744	13.32	<.0001
40-49	0.4619	0.0312	0.4007	0.5230	14.81	<.0001
50-64	0.7071	0.0335	0.6414	0.7728	21.11	<.0001
**Female (ref = male)**	0.1671	0.0209	0.1262	0.2081	8.01	<.0001
Race/ ethnicity (ref = Non-Hispanic, White)						
Non-Hispanic, Black	-0.1760	0.0517	-0.2773	-0.0748	-3.41	0.0007
Non-Hispanic, American Indian	-0.4431	0.0393	-0.5200	-0.3661	-11.28	<.0001
Hispanic	-0.2554	0.0378	-0.3295	-0.1812	-6.75	<.0001
Other/ unknown race	-0.1656	0.0498	-0.2633	-0.0680	-3.32	0.0009
Comorbidities						
Mental health (MH) (ref = No MH comorbidity)	-0.0246	0.0219	-0.0675	0.0183	-1.12	0.2614
Other substance use disorder (SUD) (ref = No SUD comorbidity)	-0.8562	0.0223	-0.8999	-0.8124	-38.34	<.0001
**Urban (ref = rural)**	-0.0236	0.0318	-0.0860	0.0387	-0.74	0.4576
Eligibility Year (ref = 2017)						
2018	-0.1233	0.0208	-0.1641	-0.0825	-5.92	<.0001
**Institution (ref = not in institution)**	-0.9093	0.1007	-1.1067	-0.7120	-9.03	<.0001

© 2023 Thomas CP et al. *JAMA Network Open.*

eTable 7. 6-Month MOUD Treatment Continuity by Disability Type
Generalized linear regression analysis for the outcome 6-month moud treatment continuity (n = 40,550 person-years for 27,688 people)

Analysis Of GEE Parameter Estimates						
**Empirical Standard Error Estimates**						
**Parameter**	**Estimate**	**Standard Error**	**95% Confidence Limits**		**Z**	**Pr > |Z|**
**Intercept**	0.3688	0.0408	0.2888	0.4489	9.03	<.0001
Disability type						
Developmental (ref = no developmental disability)	0.1183	0.0940	-0.0660	0.3025	1.26	0.2083
Physical (ref = no physical disability)	-0.1663	0.0637	-0.2912	-0.0414	-2.61	0.0091
Sensory (ref = no sensory disability)	-0.0601	0.0635	-0.1846	0.0644	-0.95	0.3441
Cognitive (ref = no cognitive disability)	-0.1188	0.0434	-0.2039	-0.0337	-2.74	0.0062
Age (ref = 18-29)						
30-39	0.3272	0.0245	0.2792	0.3753	13.35	<.0001
40-49	0.4617	0.0312	0.4006	0.5229	14.79	<.0001
50-64	0.7086	0.0337	0.6427	0.7746	21.05	<.0001
**Female (ref = male)**	0.1666	0.0209	0.1257	0.2076	7.98	<.0001
**Race/ ethnicity (ref = Non-Hispanic, White)**						
Non-Hispanic, Black	-0.1759	0.0517	-0.2771	-0.0746	-3.40	0.0007
Non-Hispanic, American Indian	-0.4436	0.0393	-0.5205	-0.3666	-11.30	<.0001
Hispanic	-0.2556	0.0378	-0.3298	-0.1815	-6.76	<.0001
Other/ unknown race	-0.1666	0.0499	-0.2643	-0.0689	-3.34	0.0008
Comorbidities						
Mental health (MH) (ref = No MH comorbidity)	-0.0272	0.0219	-0.0702	0.0157	-1.24	0.2135
Other substance use disorder (SUD) (ref = No SUD comorbidity)	-0.8551	0.0223	-0.8989	-0.8113	-38.27	<.0001
**Urban (ref = rural)**	-0.0249	0.0318	-0.0873	0.0375	-0.78	0.4336
Eligibility Year (ref = 2017)						
2018	-0.1230	0.0208	-0.1638	-0.0822	-5.91	<.0001
**Institution (ref = Not in Institution)**	-0.9138	0.1007	-1.1111	-0.7166	-9.08	<.0001

© 2023 Thomas CP et al. *JAMA Network Open.*

eTable 8. Likelihood of Receiving Buprenorphine Versus Methadone by Disability Status
Generalized linear regression analysis for the outcome buprenorphine treatment, among those receiving buprenorphine or methadone; N = 74,347 person-years for 41,393 people

Analysis Of GEE Parameter Estimates						
Empirical Standard Error Estimates						
Parameter	Estimate	Standard Error	95% Confidence Limits		Z	Pr > |Z|
**Intercept**	-0.1019	0.0360	-0.1724	-0.0313	-2.83	0.0047
**Any disability (ref = No disability)**	-0.0369	0.0255	-0.0869	0.0131	-1.45	0.1483
Age (ref = 18-29)						
30-39	-0.2342	0.0189	-0.2714	-0.1971	-12.36	<.0001
40-49	-0.5442	0.0237	-0.5907	-0.4977	-22.94	<.0001
50-64	-0.9432	0.0251	-0.9923	-0.8941	-37.65	<.0001
**Female (ref = male)**	-0.1922	0.0160	-0.2235	-0.1609	-12.04	<.0001
**Race/ ethnicity (ref = Non-Hispanic, White)**						
Non-Hispanic, Black	-0.3130	0.0384	-0.3884	-0.2377	-8.14	<.0001
Non-Hispanic, American Indian	0.0926	0.0306	0.0326	0.1525	3.03	0.0025
Hispanic	0.2293	0.0295	0.1714	0.2872	7.77	<.0001
Other/ unknown race	0.1914	0.0383	0.1163	0.2666	4.99	<.0001
**Comorbidities**						
Mental health (MH) (ref = No MH comorbidity)	0.5772	0.0164	0.5450	0.6093	35.18	<.0001

© 2023 Thomas CP et al. *JAMA Network Open.*

eTable 9. Likelihood of Receiving Buprenorphine Versus Methadone by Disability Type
Generalized linear regression analysis for the outcome buprenorphine treatment, among those receiving buprenorphine or methadone; N = 74,347 person-years for 41,393 people

Analysis Of GEE Parameter Estimates						
Empirical Standard Error Estimates						
Parameter	Estimate	Standard Error	95% Confidence Limits		Z	Pr > |Z|
**Intercept**	-0.1013	0.0360	-0.1719	-0.0308	-2.82	0.0049
**Disability type**						
Developmental (ref = no developmental disability)	-0.2851	0.0743	-0.4307	-0.1396	-3.84	0.0001
Physical (ref = no physical disability)	-0.0589	0.0490	-0.1549	0.0371	-1.20	0.2291
Sensory (ref = no sensory disability)	-0.0366	0.0496	-0.1338	0.0605	-0.74	0.4600
Cognitive (ref = no cognitive disability)	0.0090	0.0341	-0.0579	0.0759	0.26	0.7920
**Age (ref = 18-29)**						
30 - 39	-0.2353	0.0190	-0.2724	-0.1981	-12.41	<.0001
40 - 49	-0.5452	0.0238	-0.5917	-0.4986	-22.95	<.0001
50 - 64	-0.9445	0.0252	-0.9939	-0.8952	-37.54	<.0001
**Female (ref = male)**	-0.1911	0.0160	-0.2224	-0.1598	-11.96	<.0001
**Race/ ethnicity (ref = Non-Hispanic, White)**						
Non-Hispanic, Black	-0.3137	0.0385	-0.3890	-0.2383	-8.15	<.0001
Non-Hispanic, American Indian	0.0933	0.0306	0.0334	0.1533	3.05	0.0023
Hispanic	0.2293	0.0295	0.1714	0.2872	7.76	<.0001
Other/ unknown race	0.1914	0.0384	0.1163	0.2666	4.99	<.0001
**Comorbidities**						
Mental health (MH) (ref = No MH comorbidity)	0.5786	0.0164	0.5464	0.6107	35.25	<.0001
Other substance use disorder (SUD) (ref = No SUD comorbidity)	0.7486	0.0170	0.7153	0.7819	44.05	<.0001
Eligibility Year (ref = 2016)						
2017	0.4644	0.0254	0.4147	0.5141	18.31	<.0001
2018	0.8349	0.0243	0.7873	0.8825	34.36	<.0001
2019	1.0815	0.0240	1.0345	1.1285	45.11	<.0001
**Urban (ref = rural)**	-0.8232	0.0262	-0.8745	-0.7719	-31.45	<.0001
**Institution (ref = Not in Institution)**	-0.0040	0.0791	-0.1591	0.1510	-0.05	0.9594

© 2023 Thomas CP et al. *JAMA Network Open.*

© 2023 Thomas CP et al. *JAMA Network Open.*
