# Supplementary material for: Quality of Opioid Use Disorder Treatment for Persons With and Without Disabling Conditions
Source: JAMA Netw Open. 2023 Mar 8;6(3):e232052. doi: 10.1001/jamanetworkopen.2023.2052 (PMC9996401; doi:10.1001/jamanetworkopen.2023.2052)
Supplement: Supplement 2. — Data Sharing Statement [file jamanetwopen-e232052-s002.pdf]

Data Sharing Statement

Thomas. Quality of Opioid Use Disorder Treatment for Persons With and Without Disabling Conditions. *JAMA Netw Open*. Published March 08, 2023.
doi:10.1001/jamanetworkopen.2023.2052

Data
Data available: No

Additional Information
Explanation for why data not available: This is patient-related information and not available for sharing. A codebook can be made available to replicate analyses.
